# Vimentin Inhibits Neuronal Apoptosis After Spinal Cord Injury by Enhancing Autophagy

**DOI:** 10.1111/cns.70200

**Published:** 2025-01-03

**Authors:** Jie Zhao, Kangzhen Chen, Tao Wang, Xianxiu Qiu, Xiaomin Zhang, Tao He, Liji Chen, Jiahong Chen, Xiaojun Cui, Hongfu Wu

**Affiliations:** ^1^ Dongguan Key Laboratory of Stem Cell and Regenerative Tissue Engineering, the First Dongguan Affiliated Hospital, School of Basic Medical Sciences Guangdong Medical University Dongguan China; ^2^ Department of Venereal Diseases and Integrated Chinese and Western Medicine and Bone Paralysis Longjiang Hospital of Shunde District Foshan China

**Keywords:** apoptosis, autophagy, neuron, spinal cord injury, vimentin

## Abstract

**Aims:**

Neuron death is caused primarily by apoptosis after spinal cord injury (SCI). Autophagy, as a cellular response, can maintain cellular homeostasis to reduce apoptosis. We aimed to investigate the effect and the mechanism of vimentin knockdown on autophagy and neural recovery after SCI.

**Methods:**

The SD rats with T10 complete transection as SCI model were used. The vimentin RNAi adenovirus was constructed and transplanted into T10 rats with total transection injury of the spinal cord, and the recovery of neurological and motor functions after SCI was evaluated by BBB score, footprint analysis, electrophysiological tests, and immunofluorescence staining. Protein and gene expression were assessed by Western blotting, CO‐IP, q‐PCR, and immunofluorescence. In addition, neuron‐like PC12 cells were infected with adenovirus to further elucidate the effect of vimentin on autophagy and the molecular mechanism of neuronal apoptosis after SCI.

**Results:**

Inhibition of SCI induced‐vimentin upregulation improved motor function, enhanced the recovery of autophagy flux, and reduced neuronal apoptosis. Notably, this may be related to the formation of vimentin‐14‐3‐3‐Beclin1 complex and PI3K class III complex.

**Conclusion:**

Our results suggest that inhibition of vimentin expression may enhance autophagy and anti‐apoptosis in neurons after SCI by affecting the formation of the vimentin‐14‐3‐3‐Beclin1 complex, thereby promoting neuronal recovery.

## Graphical abstract

After SCI, vimentin knockdown increased the amount of free state Beclin1 by inhibiting the binding among Beclin1, 14‐3‐3 and vimentin, promoted the formation of PI3K class III complexes, and increased the level of autophagy in neuronal cells after SCI, while inhibiting neuronal apoptosis.

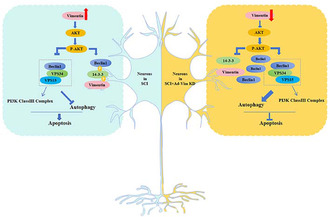

## 1 Introduction

Spinal cord injury (SCI) is a type of damage to the structure and function of the spinal cord due to a variety of causes, severely impairing the upper and lower nerve conduction pathways and leading to varying degrees of motor and sensory dysfunction. SCI includes both primary and secondary injury, which together result in irreversible neuronal damage [1, 2]. During SCI, neuron death is the main cause of neurological deficits, ultimately leading to poor functional recovery in patients after SCI. Therefore, finding a way to effectively inhibit neuronal apoptosis and reestablish its connection with the original target after SCI may be a promising strategy for the treatment of SCI.

Autophagy is a complex process whereby cells engulf and degrade their structural proteins and damaged organelles, which regulates the balance between cell survival and death under stressful conditions. It's a way to restore cellular homeostasis [2]. In addition, several studies have shown that autophagy plays a “double‐edged sword” role in SCI. In the early stage of SCI, increasing autophagy helps to accelerate the degradation of damaged mitochondria and harmful proteins by lysosomes, creating a favorable environment for neural regeneration of surviving neurons and reducing the level of apoptosis of spinal cord neurons [3]. When the SCI is more severe, the autophagic flow is disturbed, and the degradation of autophagic substrates is difficult, and the promotion of autophagy at this time leads to the difficulty of degradation of autophagic substrates with neurotoxicity and stress overload, which further leads to apoptotic necrosis of neurons [4, 5, 6]. Therefore, regulating autophagy so that it inhibits apoptosis within a controlled range and thus promotes the recovery of neurological function after SCI is an important idea in current research.

Vimentin, a member of the intermediate filament protein family, plays important roles in a variety of biological processes, such as cell survival, induction of apoptosis, inhibition of differentiation, and promotion of cell invasion and migration [7, 8, 9]. Previous studies on vimentin have mainly focused on cancer, vimentin expression can over‐promote EMT and participate in the adhesion migration invasion of tumor cells and their associated endothelial cells and macrophages [10, 11]. Previous studies have shown that inhibition of vimentin and GFAP expression after SCI is beneficial to reduce glial scar formation and promote the recovery of neurological function after SCI [12, 13]. Another research indicated that vimentin‐deficient neural stem cells in response to upregulated levels of aggregated proteins by upregulating autophagy, thereby restoring protein homeostasis [14]. However, the role of vimentin in the neuronal survival and autophagy remains unclear.

Herein, with a SCI model in mice, we found that neuronal vimentin expression was upregulated in the injured spinal cord. Inhibition of vimentin by RNAi adenovirus transfection could enhance autophagy and anti‐apoptosis in neurons after SCI by affecting the formation of the vimentin‐14‐3‐3‐Beclin1 complex, thereby promoting neuronal recovery.

## 2 Materials and Methods

### 2.1 Construction of Adenovirus Vectors

To evaluate the role of vimentin knockdown in SCI, we constructed a vimentin adenoviral expression vector for transfection of PC12 cells in vitro or for in vivo injection into animals. The adenoviruses used in the experiments were constructed and synthesized by Jikai Gene Company, including Vim‐RNAi‐101945 (titer: 4 × 10^10^ PFU/mL), Vim‐RNAi‐101946 (titer: 3 × 10^10^ PFU/mL), Vim‐RNAi‐101947 (titer: 2 × 10^10^ PFU/mL), and a negative control vector (titer: 5 × 10^10^ PFU/mL).

### 2.2 Animals

A total of 80 specific pathogen‐free (SPF) adult female Sprague–Dawley rats were used in this experimental study, obtained from the Experimental Animal Center of Southern Medical University in Guangzhou, China. All laboratory procedures involving animals were conducted by guidelines outlined in the “Guide for the Care and Use of Laboratory Animals.”

### 2.3 SCI Rat Model and Grouping

Anesthesia was administered via intraperitoneal injection of 1% sodium pentobarbital (40 mg/kg) based on body weight. Once deep anesthesia was achieved (indicated by regular respiration in rats, loss of corneal reflexes, muscle relaxation, and unresponsiveness to stimulation, among other signs), the surgical site on the dorsal area was prepared. The rats were positioned prone on the operating table, and a longitudinal incision of approximately 1.5 cm was made along the dorsal midline. This incision involved carefully dissecting through the fascia and paravertebral muscles to expose the spinal segments from T9 to T11. A laminectomy was performed at T10 to reveal the spinal cord, which was then fully transected using a scalpel. Following this, the animals were treated according to their respective groups, with layered sutures applied for hemostasis in the muscles, fascia, and skin. After the surgery, the rats were placed on an electric blanket set to maintain a temperature of 37ºC. Once they regained consciousness, they were transferred to individual cages for recovery. The rats' bladders were manually emptied three times a day to aid in urination. A 1 mL injection of a penicillin–streptomycin mixture (10,000 U/mL) was administered via intraperitoneal injection within 1–3 days after surgery to prevent postoperative infections. The animals were randomly assigned to one of the following three groups, each consisting of 20 rats: (1) the sham‐operated group (sham group); (2) the PBS control group (SCI + PBS group); (3) the negative control group (SCI + Ad‐NC group); and (4) the Vimentin RNAi adenovirus‐infected group (SCI + Ad‐Vim KD group).

### 2.4 Real‐Time Quantitative PCR Assay

Total RNA was extracted from rat tissues at T9–T11 level or cells using Trizol reagent (Invitrogen, Carlsbad, CA, USA). The purity and concentration of total RNA in each sample were assessed by determining the absorbance using UV–Vis spectrophotometry. Subsequently, cDNA was synthesized using reverse transcriptase following the instructions provided in Takara's TB Green Premix reagent kit. Real‐time fluorescence quantitative PCR was conducted using SYBR Premix Ex Taq (RR420A, TAKARA). The expression level of GAPDH was used as an internal reference, and three replicate wells were set up for each reaction. All primers used in this experiment were provided by Bioengineering Ltd. The primers used included the following: vimentin forward, 5′‐TCAGACAGGATGTTGACAAT‐3′ and reverse, 5′‐GACATGCTGTTCCTGAATCT‐3′; GAPDH primer: forward, 5′‐CTGGAGAAACCTGCCAAGTATG‐3′ and reverse, 5′‐GGTGGAAGAATGGGAGTTGCT‐3′.

### 2.5 Behavioral Analysis

The functional motor recovery of rats was evaluated using the BBB scale at 1, 2, 3, 4, 5, 6, 7, and 8 weeks after surgery. The final score for each rat was determined by averaging the values provided by the two researchers, ensuring objectivity in the scoring process.

### 2.6 Footprint Analysis

To assess the recovery of motor function after SCI, the rats were put to run along a white paper, which was placed inside a wooden box with specific dimensions (an inner diameter of 50 cm, 8.5 cm in width, and 10 cm in height). Each hindlimb of the rats was brushed with red ink, and analysis of motor function recovery in rats was based on footprints.

### 2.7 Neuroelectrophysiology Assay

The spinal cord electrical signals of each group of animals were detected using the MedLab6 Bio‐signal Acquisition and Processing System. Briefly, after 8 weeks of SCI, rats were anesthetized by intraperitoneal injection of 1% sodium pentobarbital (40 mg/kg), fixed in the prone position, and the T5‐L2 spinal cord was exposed. The T5‐L2 spinal cord was exposed, and the spinal cord was moistened with saline. The spinal cord was moistened with saline. The stimulating electrode was gently hooked onto the center of injury (T10). The stimulating electrode was gently hooked onto the spinal cord about 1 cm above T10, and the recording electrode was immediately attached to the spinal cord about 1 cm below T10.

### 2.8 Western Blot Assay

Tissue samples (1 cm above and below the injury center) and cell samples were lysed with a protein lysate: RIPA, 100 mM PMSF, 100 mM Protein Phosphatase Inhibitor. Centrifuged at 12,000 rpm for 30 min, and extracted with 5xloading buffer boiled at 100°C for 10 min. The protein samples were separated using 10% or 12% (w/v) SDS‐PAGE and after blocking with 5% (w/v) skim milk for 2 h at room temperature, and then the membranes were incubated with primary antibodies overnight at 4°C. The membrane was incubated with HRP Goat Anti‐Mouse/Rabbit IgG (1:10,000) antibody for 1 h at room temperature. The primary antibodies included rabbit anti‐vimentin (Cat.#5741S, Cell Signaling Technology, 1:1000), rabbit anti‐LC3B (ab192890, abcam, 1:1000), rabbit anti‐p62 (Cat.#23214S, Cell Signaling Technology, 1:1000), rabbit anti‐Bcl‐2 (ab182858, abcam, 1:1000), rabbit anti‐Bax (Cat.#CPA3797, Bioworld, St. 1:1000), rabbit anti‐caspase3 (Cat.#9662, Cell Signaling Technology, 1:1000), mouse anti‐caspase9 (Cat.#9508, Cell Signaling Technology, 1:1000), rabbit anti‐PI3K (Cat.# 4263S, Cell Signaling Technology, 1:1000), rabbit anti‐AKT (Cat.#9272, Cell Signaling Technology, 1:1000), rabbit anti‐P/AKT (Cat.#9271, Cell Signaling Technology, 1:1000), rabbit anti‐Beclin1 (NB500, NOVUS, 1:1000), mouse anti‐NeuN (Millipore, 1:1000), mouse anti‐GAPDH (Cat.#60004‐1, Proteintech Group In, 1:1000), were incubated overnight at 4°C. Detection was performed using ECL chemiluminescence, and images were analyzed for protein bands for gray values and normalized protein levels to those of GAPDH. Data S1 show the full unedited blot images.

### 2.9 Nissl Staining

Nissl staining was employed to examine the pathological changes in neurons following SCI. Briefly, after perfusion of rats with 0.9% NaCl followed by 4% PFA, the spinal cord was immersed in 4% PFA for 24 h and transferred to 30% sucrose solution until immersion. Subsequently, T9–T11 thoracic spinal cord segments were cut into 12 μm‐thick sections using a cryosectioner. For the sections, the sections were immersed in 1 × PBS for 10–15 min. The sections were incubated with the staining solution for 10 min at room temperature, and then rinsed with double‐distilled water and 95% ethanol. They were subsequently dehydrated in 100% ethanol, cleared in xylene and then covered with neutral resin.

### 2.10 Immunofluorescence Staining

After perfusion of rats with 0.9% NaCl followed by 4% PFA, the spinal cord was immersed in 4% PFA for 24 h and transferred to 30% sucrose solution until immersion. Subsequently, T9–T11 thoracic spinal cord segments were cut into 12 μm‐thick sections using a cryosectioner. For staining of spinal cord tissue sections, the sections were thawed for 30 min at room temperature, embedding agent was removed by immersing in 1 × PBS for 10–15 min, incubated with proteinase K (Sigma‐Aldrich, 1:2000) for 10 min at 37°C, and washed three times with 0.1 mM PBS containing 0.1% Triton X‐100. This was followed by blocking for 1 h at room temperature using 5% BSA. The sections were then incubated with primary antibodies at 4°C overnight. The sections were subsequently washed three times with PBS and then incubated with the appropriate secondary antibody for 1 h at room temperature. The fluorescent primary antibodies included: rabbit anti‐LC3B (ab192890, Abcam, 1:500), mouse anti‐NeuN (Millipore 1:500), rabbit anti‐Beclin1 (NOVUS, 1:500), rabbit anti‐vimentin (Cat.#5741S, Cell Signaling Technology, 1:500). The secondary antibodies included: anti‐rabbit IgG H&L (Alexa Fluor 488, 1:500), goat anti‐mouse IgG H&L (Alexa Fluor 568, 1:500).

For cell staining, briefly, cells were rinsed 3 times with PBS, followed by fixation with 4% PFA for 20 min. Subsequently, the cells were permeabilized with 0.1% Triton X‐100 for 10 min at room temperature, blocked with 5% BSA for 1 h and incubated with primary antibody overnight at 4°C. Cell nuclei were stained with DAPI (Cell Signaling Technology, 1:5000), and images were acquired using a fluorescence microscope.

### 2.11 Cell Culture

Rat pheochromocytoma cells (PC12 cells) were originally obtained from the Chinese Academy of Sciences (Shanghai, China). The PC12 culture medium consisted of Dulbecco's modified Eagle's medium (DMEM, Gibco), 10% fetal bovine serum (FBS, Gibco), and 1% penicillin/streptomycin (100 U/mL, Sigma Aldrich, St. Louis, MO, USA) at 37°C with 5% CO_2_.

### 2.12 Cell Grouping and Transfection

Cells were randomly divided into the following groups: control group (no adenovirus transfection), Ad‐NC group (transfected with negative control vector), and Ad‐Vim KD group (transfected with vimentin knockdown adenovirus). Cell transfection was performed in 6‐well plates with a density of 1.5 × 10^4^/mL of inoculated cells per well, using DMEM medium containing 10% FBS. Adenoviral infection could be performed when cell fusion reached 60%. After discarding the original medium, 1 mL of prepared infection medium (1 mL of DMEM medium containing 10% fetal bovine serum but no double antibody to dilute adenovirus) was added using the 1/2 small volume infection method. After 8–12 h of infection, the infection medium was discarded, and the culture was continued with 2 mL of fresh complete medium. Real‐time fluorescence quantitative polymerase chain reaction (qRT‐PCR) was used to detect the transfection efficiency of the vimentin knockdown adenovirus vector in PC12 cells.

### 2.13 Cell Migration Assay

PC12 cells were seeded in 6‐well plates, cultured normally or infected with adenovirus for 48 h before the wound healing assay. Draw a straight line through the center of the circle with a 200 μL muzzle. Make sure the muzzle is vertical, and replace it with a new one for each well. The old culture was then discarded, cells were gently washed once with 1 × PBS attached to a wall six‐well plate to remove scratched cells, and medium containing 1% fetal bovine serum was replaced following (to reduce the effect of cell proliferation). The photographs were taken at different time points: 0, 12, and 24 h.

### 2.14 Cell Proliferation Assay

Cell proliferation analysis was performed using Edu staining 48 h after adenoviral transfection. Briefly, cells were transfected for 24 h, incubated with Edu working solution for 4–5 h, fixed with 4% paraformaldehyde, incubated at room temperature for 30–60 min, and stained with Click reaction solution and 1 × Hoechst 33342 to avoid light. Sealed with anti‐fluorescent bursting agent. Photographed using a live cell imager, proliferating cells showed red fluorescence and total cells showed blue fluorescence. The proportion of proliferating cells to total nuclei was calculated as the cell proliferation rate. The proliferation index was the percentage of Edu‐positive cells in each group.

### 2.15 LC3B Turnover Assay

An appropriate number of cells (approximately 3.0 × 10^4^ cells/well) were seeded in 6‐well plates. The cells were incubated overnight and infected with adenovirus. After 8–12 h of infection, the infected medium was removed and replaced with 2 mL fresh complete medium for further culture. After 24 h of adenovirus infection, complete medium containing 20 μmol/L chloroquine was replaced and cultured for another 24 h. Cell proteins were collected and detected by Western blot.

### 2.16 Co‐Immunoprecipitation

Cellular proteins were collected using IP protein lysis solution; cells were scraped from the culture dish with a pre‐cooled cell scraper and transferred to 1.5 mL EP tubes for 1 h at 4°C with slow shaking. Afterwards, centrifuge at 4°C, 12,000 rpm for 20 min and collect the supernatant. For each subgroup, 500 μg of protein was mixed with 2–3 μg of antibody (Beclin1, Santa cruz) and incubated overnight at 4°C with moderate rotation, and then incubated for an additional 2 h after the addition of 30 μL of protein A/C magnetic beads. The magnetic beads were collected with a magnetic rack and washed five times with lysis buffer and resuspended in 1 × SDS sample buffer. After boiling for 10 min, the immunoprecipitates were subjected to SDS‐PAGE and standard immunoblotting procedures.

### 2.17 mCherry‐GFP‐LC3 Assay

Ad‐mCherry‐GFP‐LC3B is an adenovirus expressing the mCherry‐GFP‐LC3B fusion protein, which can be used to detect autophagic flux after infection of cells. Cells were inoculated in 12‐well plates according to 8 × 10^3^/well and adenoviral infection was performed according to an MOI value of 50, and the medium was changed for 8–12 h. After 48 h of transfection, the cells were placed under a laser confocal microscope for observation. ImageJ analyzed the images for fluorescence intensity. Yellow spots (formed by overlap between red and green) indicate autophagosomes, while red spots indicate autolysosomes.

### 2.18 Statistical Analysis

In this study, all data were analyzed using SPSS 22.0 or GraphPad Prism 5.0 statistical software. The Shapiro–Wilk normality test was employed to confirm that the data followed a normal distribution. Measurement data with normal distribution and homogeneous variances are expressed as mean ± standard deviation. Statistical methods were based on one‐way ANOVA and independent samples t‐test between three and more groups. *p* < 0.05 represents a statistical difference.

## 3 Results

### 3.1 The Expression of Vimentin Is Upregulated After SCI

**FIGURE 1:**
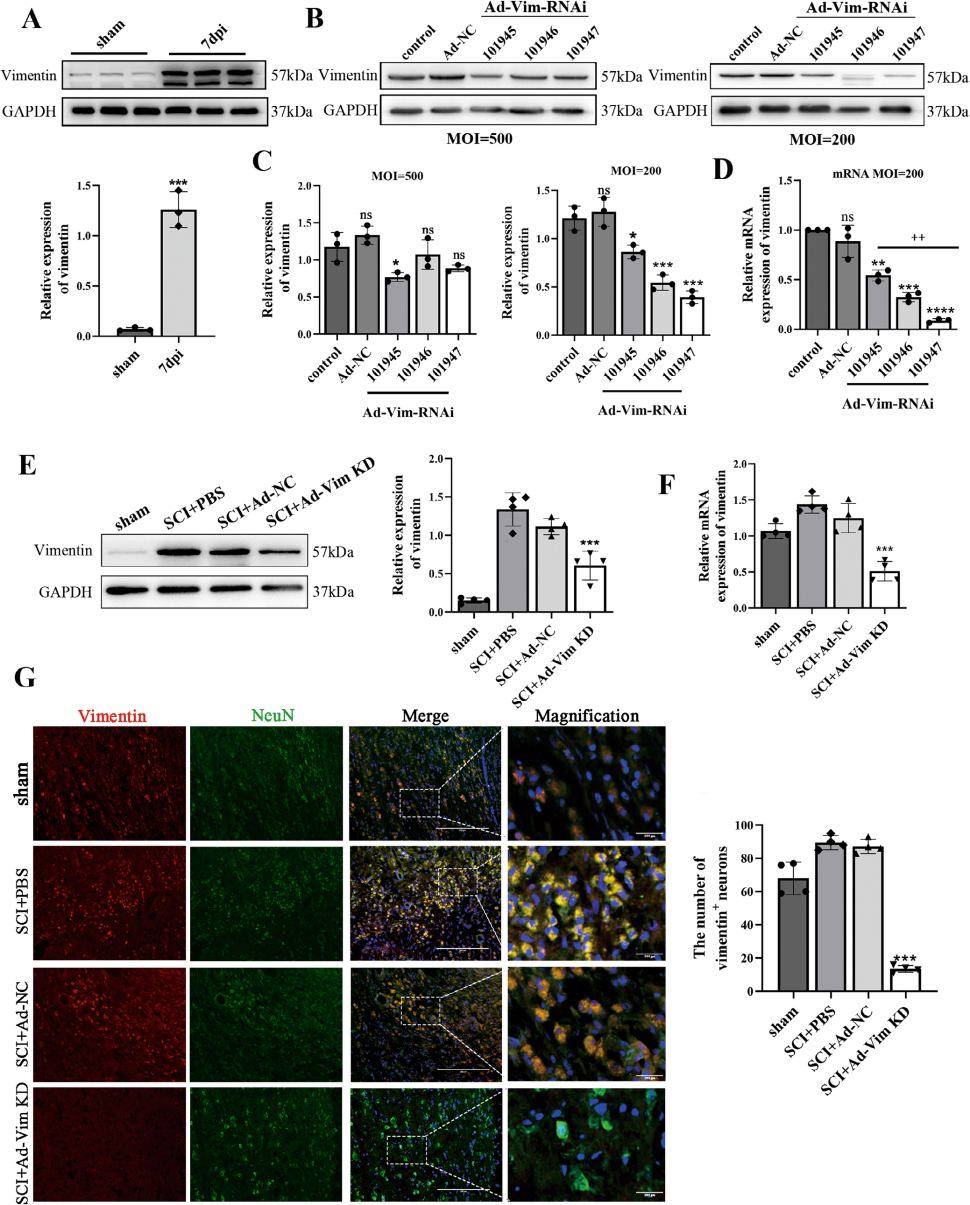
Vimentin RNAi adenovirus effectively inhibits vimentin expression in PC12 cells and rats. (A) Representative western blot results of vimentin expression of sham group and SCI group at 7 days after SCI. (B) western blot detection of vimentin protein expression at MOI = 500 and MOI = 200. (C) Quantitative analysis of vimentin protein expression at MOI = 500 and MOI = 200. (D) qRT‐PCR to verify the expression of vimentin RNAi adenoviral infection of PC12 cells at MOI = 200 at the level of vimentin mRNA. *p < 0.05, ***p < 0.001, versus control group, ^++^ *p* < 0.01, versus Ad‐Vim‐RNAi 101945 group; Scale bar = 400 μm (A). (E) western blot detection of knockdown efficiency of vimentin RNAi adenovirus and relative quantitative analysis of vimentin protein. (F) Relative quantitative analysis of vimentin mRNA. (G) Representative images showing immunofluorescence staining of NeuN/Vimentin co‐localization at lesions after SCI (Scale bar = 200 μm). Enlarged image of the box area (white box), scale bar = 200 μm. *p < 0.05, **p < 0.05, ***p < 0.001, versus SCI + PBS group.

Vimentin plays a crucial role as an integral component in maintaining cellular homeostatic. Previous reports have indicated that vimentin is typically expressed at low level in neural stem cells under normal conditions. However, its expression significantly increases following neurological injuries [15, 16]. In this study, we initially investigated alterations in vimentin expression before and after SCI in rat. Compared with the sham group, vimentin was differentially upregulated after SCI (Figure 1A). This observation suggests a potential causal relationship between vimentin expression and the recovery process after SCI.

### 3.2 Vimentin Knockdown Promoted Functional Recovery After SCI

To investigate the role of vimentin in SCI, we evaluated the efficacy of adenovirus‐mediated knockout both in vitro and in vivo. We detected the mRNA and protein levels of vimentin in cells transfected for 48 h and in spinal cord tissue at 14 days post‐infection (dpi). The results showed that adenovirus effectively downregulated vimentin expression both in vitro and in vivo (Figure 1B–G). Based on these findings, we proceeded with the following research.

**FIGURE 2:**
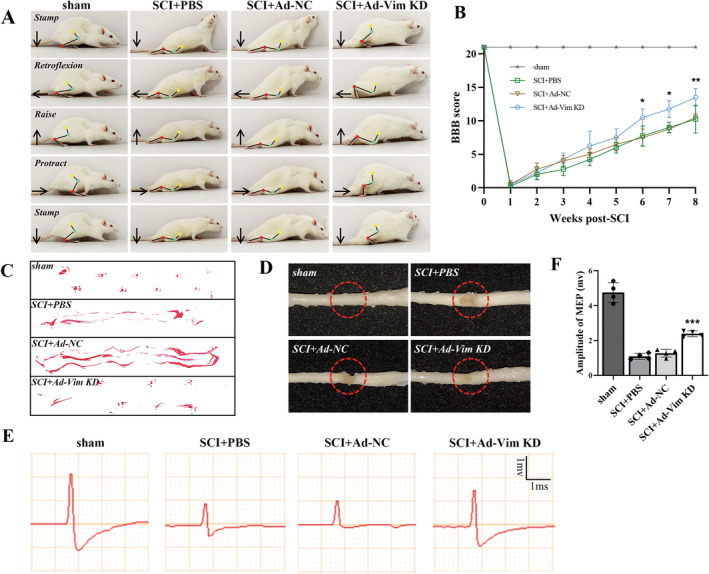
Vimentin knockdown promotes functional recovery after SCI. (A) Representative images of rat hindlimb behavior at 8 weeks postoperatively for each group; (B) BBB scores of the sham, SCI + PBS, SCI + Ad‐NC, and SCI + Ad‐Vim KD groups at 1 day before SCI and weekly for 8 weeks after SCI; data are shown as the mean ± SD (*n* = 4/group); (C) Footprint analysis images of each group at 8 weeks after SCI. (D) Representative spinal cord specimens in each group at 8 weeks postoperatively. (E) Electrophysiological activity and the amplitude of electromyography in each group at 8 weeks postoperatively; *p < 0.05, **p < 0.01, ***p < 0.001, versus SCI + PBS group.

We next assess improvement in hind limb locomotor joint movements (Figure 2A). Further, using BBB scale test and footprint analysis, we found that compared with SCI groups, vimentin knockdown improved functional recovery (Figure 2B,C). We also took the visual images of morphology of spinal cords, as shown in Figure 2D. The spinal cord in the SCI group showed significant defects and atrophy, while that in the SCI+Ad‐Vim KD group showed a relatively milder degree of spinal cord atrophy and smaller defects. Consistently, by electrophysiological test, vimentin‐treated groups showed the higher maximum amplitude of spinal evoked potential of SCI when compared with SCI group (Figure 2E,F). Collectively, these results suggested that vimentin knockdown could enhance nerve conduction in SCI rats, exerting a therapeutic effect on the functional recovery from SCI.

### 3.3 Vimentin Knockdown Inhibits Neuronal Apoptosis After SCI

**FIGURE 3:**
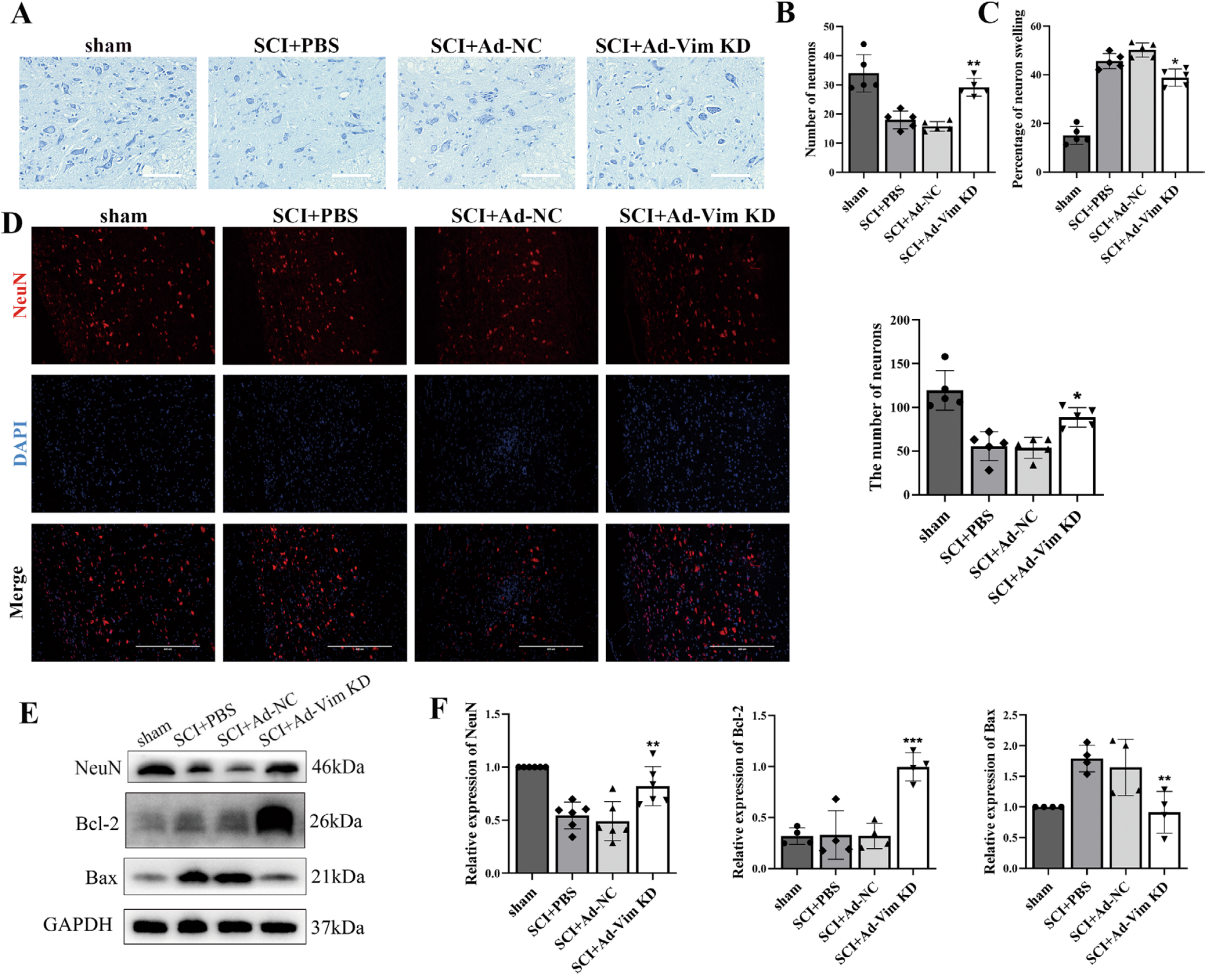
Vimentin knockdown inhibits neuronal apoptosis after SCI. (A) Representative images showing Nissl staining of spinal cord tissue and (B, C) quantitative analysis of the results. Scale bar = 100 μm. (D, E) Representative images showing the effect of vimentin on neuronal survival by NeuN immunofluorescence staining and quantitative analysis of the results. The fluorescence indicator is Alexa Fluor 568 (red) for NeuN. Cell nuclei were stained with DAPI (blue), Scale bar = 400 μm. (E, F) Representative Western blot results and relative quantitative data analysis of neuronal marker NeuN and apoptosis‐related proteins Bax and Bcl‐2. *p < 0.05, **p < 0.01, versus SCI + PBS group.

Nissl staining was performed to examine the structure of the spinal cord tissues. The staining showed that compared with Sham group SCI groups caused neuronal loss, while the number of neurons of SCI + Ad‐Vim KD group rats was greater than that of SCI and SCI + PBS group rats, and significantly decreased neuronal swelling (Figure 3A–C). Furthermore, we conducted NeuN immunofluorescence staining on the sections of injured tissue to identify NeuN‐positive neurons. The result showed that there were more fluorescence signals in vimentin knockdown group when compared with SCI group (Figure 3D,E). And as revealed by western blot analysis, the expression level of NeuN protein was increased in SCI + Ad‐Vim‐KD group. Moreover, SCI groups significantly upregulated the expression of Bax, but Bax expression was significantly reduced after vimentin knockdown, while Bcl2 expression was significantly increased (Figure 3G,H). These findings indicate that the restorative effect of vimentin after SCI is partly attributed to the inhibition of neuronal apoptosis.

### 3.4 Vimentin Knockdown Enhances Neuronal Autophagy After SCI

**FIGURE 4:**
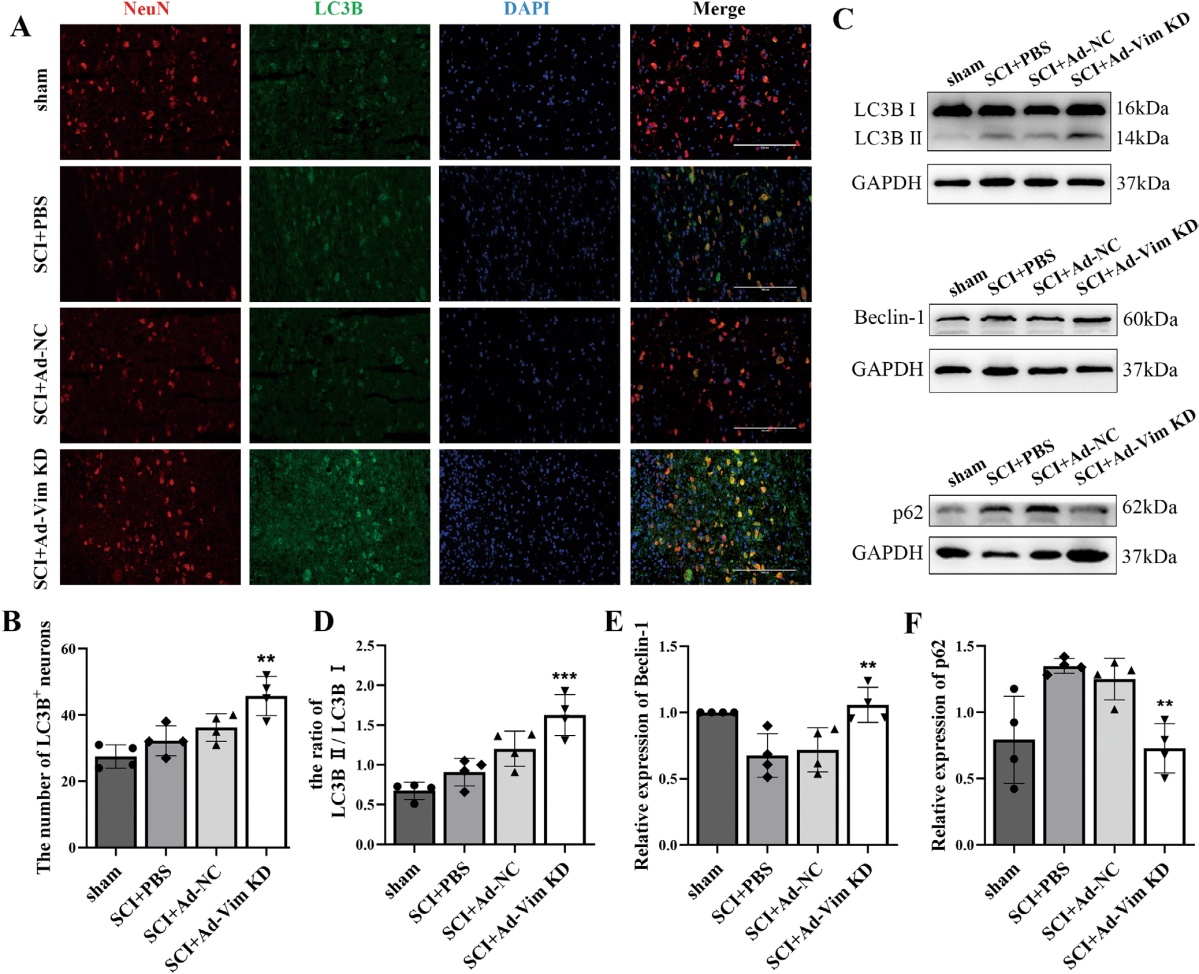
Vimentin knockdown enhances neuronal autophagy in vivo after SCI. (A) Representative images showing immunofluorescence staining of NeuN/LC3B co‐localization at lesions after SCI (Scale bar = 200 μm) (B) Percentage of LC3II‐positive neuron numbers at spinal cord lesions in each group; (C) Representative western blot results of autophagy‐related proteins LC3B, Beclin‐1, and P62, GAPDH were used as an internal control and (D–F) relative quantitative analysis. *p < 0.05, **p < 0.01, ***p < 0.001, versus SCI + PBS group.

To further explore whether the mechanism of vimentin effect is related to the regulation of autophagy. The level of the LC3 in neurons was assessed by NeuN (green)/LC3 (red) double staining. Compared with the SCI + PBS group, the percentage of LC3B‐positive neurons increased significantly in the SCI + Ad‐Vim KD group (Figure 4A,B). Furthermore, the western blot analysis results indicated that LC3BII expression significantly increased in the SCI + Ad‐Vim KD group compared with the SCI + PBS group. Moreover, the expression of autophagy‐related protein Beclin1 was higher in the SCI + Ad‐Vim KD group than in the SCI + PBS group. Accumulation of p62 is constantly used as a marker of impaired autophagy, western blot analysis of P62 showed SCI‐induced p62 accumulation, while vimentin knockdown reduced p62 expression (Figure 4C–F). In brief, under normal conditions, the autophagy level of spinal cord neurons is low, and after SCI, vimentin knockdown not only increases the expression of autophagosome‐related markers but also reduces the burden of autophagic substrates, which may be due to the increase of overall autophagic activity level after SCI caused by vimentin.

### 3.5 In Vitro Vimentin Knockdown Attenuates Neuronal Apoptosis by Activating Autophagy

**FIGURE 5:**
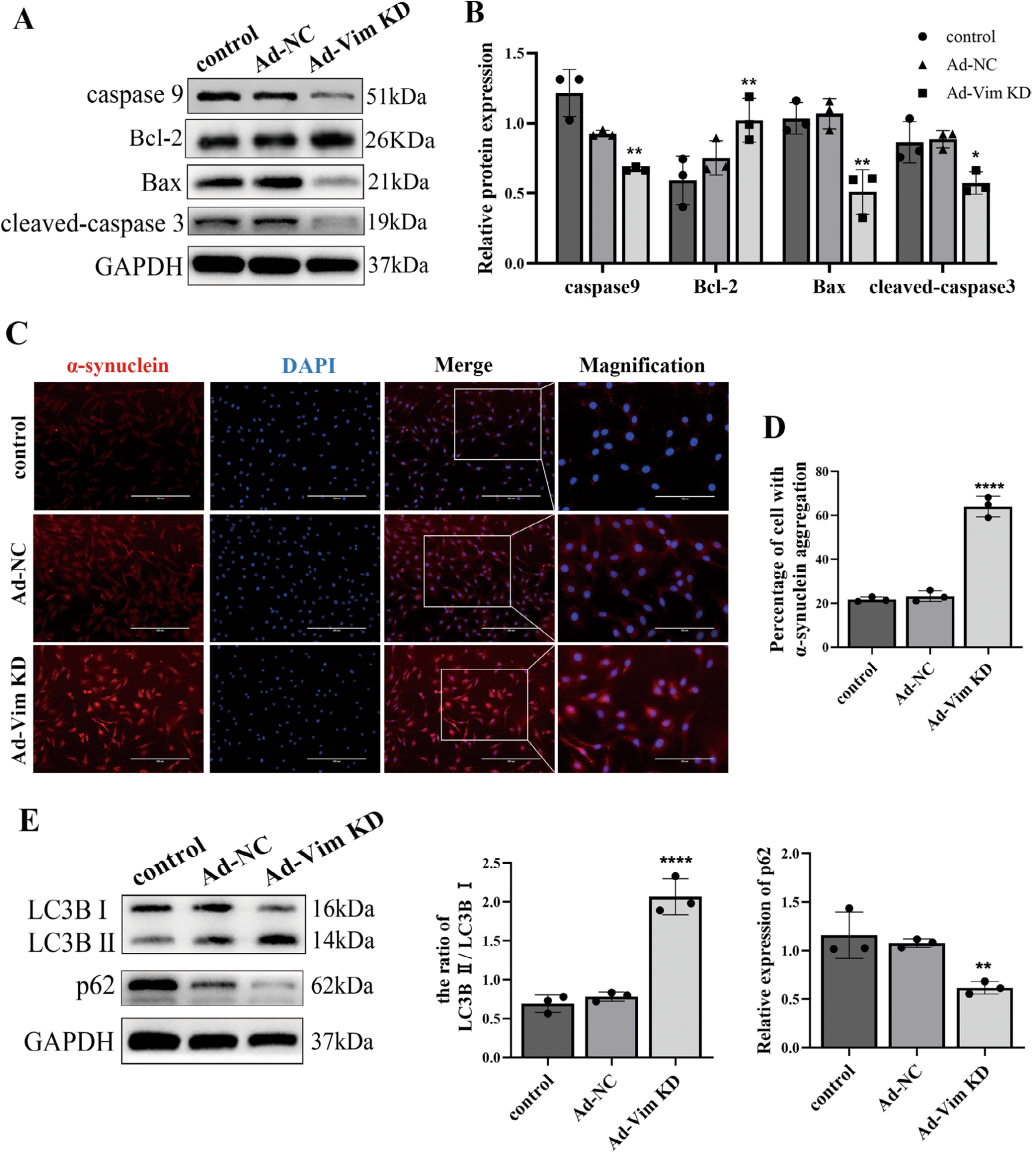
Vimentin knockdown activates autophagy and attenuates neuronal apoptosis in vitro. (A) Western blot analysis and (B) relative quantitative analysis of apoptosis‐related proteins caspase9, Bcl‐2, Bax, cleaved‐caspase3, GAPDH were used as an internal control. (C) Immunofluorescence staining of α‐synuclein in PC12 cells; (D) Statistics of the percentage of α‐synuclein positive cells; scale bar = 200um (Merge), scale bar = 400um (Magnification). (E) Western blot analysis and relative quantitative analysis of autophagy‐related proteins LC3B, P62, and GAPDH were used as an internal control. *p < 0.05, **p < 0.01, ****p < 0.0001, versus control group.

To determine the effects of vimentin on neuronal apoptosis, we transfected PC12 cells with vimentin RNAi adenovirus. As shown in Figure 4A, the western blot analysis results showed that after vimentin knockdown, the expression of caspase9, Bax, and cleaved‐caspase3 was decreased significantly. Moreover, the expression of Bcl‐2 was higher in the Ad‐Vim KD group (Figure 5A,B). Apoptosis is the opposite process of cell proliferation; the two are both opposing and united in a coordinated manner [17]. We next performed EDU staining after 48 h of transfection to confirm cell proliferation; the Ad‐Vim KD group showed lower cell proliferation rate when compared with the control group (Figure S1A,B). The wound‐healing assay was used to detect the cell migration abilities after vimentin knockdown. As demonstrated in Figure S1C,D, compared with the control group, the Ad‐Vim KD group filled less area after 12 and 24 h relative to 0 h. These results showed that vimentin knockdown inhibited cell proliferation and migration. The reduction of neuronal apoptosis caused by inhibition of vimentin expression was not through affecting proliferation.

SCI leads to the aggregation of α‐synuclein, and increasing autophagy levels enables the removal of protein aggregates from neurons. We performed immunofluorescence staining, and the results showed that more α‐synuclein accumulations were formed in the Ad‐Vim KD group when compared with the control group (Figure 5C,D). Additionally, western blotting analysis showed that compared with the control group, vimentin knockdown significantly promoted the autophagy‐related protein LC3BII expression, but decreased the expression of P62 (Figure 5E). These results showed that vimentin knockdown could inhibit apoptosis by increasing the effect of autophagy rather than cell proliferation.

### 3.6 Vimentin Knockdown Favors Autophagosome Formation in Pre‐Autophagic Periods

**FIGURE 6:**
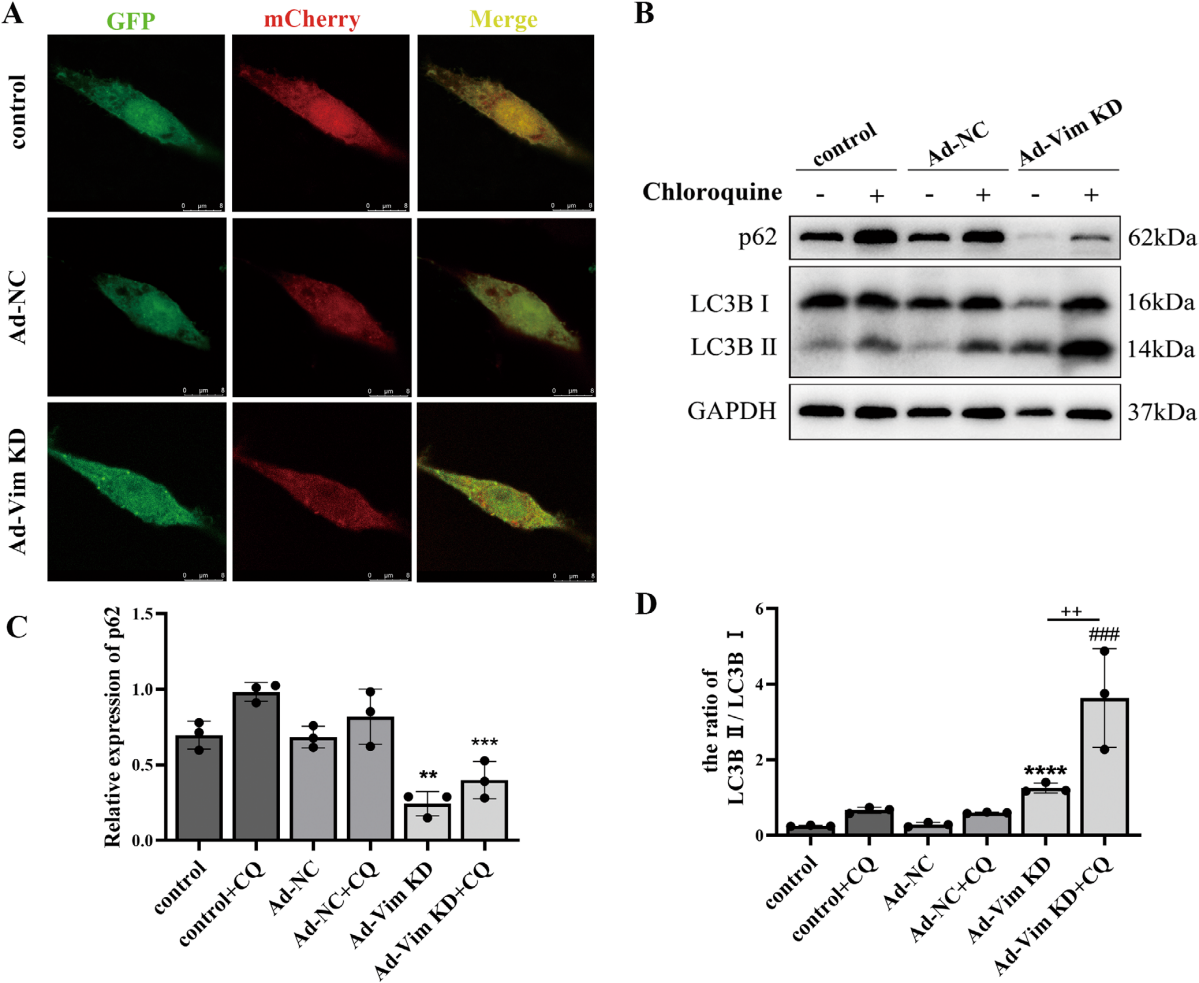
Vimentin knockdown favors autophagosome formation during pre‐autophagy. (A) Representative fluorescence map of Ad‐mCherry‐GFP‐LC3B transfected PC12 cells. Autophagosomes are labeled by red and green fluorescence (yellow puncta); Scale bar = 8 μm. (B–D) Western blot analysis and relative quantitative analysis of P62, LC3B in the presence and absence of chloroquine, GAPDH was used as an internal control. **p < 0.01, ***p < 0.001, ****p < 0.0001, versus control; ^###^ *p* < 0.001, versus control + CQ group; ^++^ *p* < 0.01, versus Ad‐Vim KD.

To further understand the effects of vimentin on autophagy, Ad‐mCherry‐GFP‐LC3B was transfected into PC12 cells and monitored the autophagic flux. When autophagy occurred in PC12 cells, autolysosomes were formed and at this time, GFP would be quenched by the acidic environment of the lysosome, while mCherry fluorescence is not affected by acidic environment. Autolysosomes can be visualized as red LC3B foci. When the autophagosome‐lysosome fusion is blocked or the lysosomal environment PH value increases, both mCherry and GFP can be detected resulting in yellow LC3B puncta. As anticipated, in the control and Ad‐NC group, mCherry‐GFP‐LC3B was mainly dispersed into the cytoplasm with yellow fluorescence. While, in the Ad‐Vim KD group, mCherry‐GFP‐LC3B was present as yellow puncta (Figure 6A).

Our data showed that vimentin knockdown could inhibit apoptosis in neurons. To further explore the relationship between autophagy and apoptosis induced by vimentin, we used a classical autophagy inhibitor chloroquine (CQ) to block autophagic flux. Compared with the control group, we found that the conversion of LC3‐I to LC3‐II was significantly increased in the Ad‐Vim KD group after chloroquine treatment (Figure 6B–D), suggesting that vimentin knockdown promotes the pre‐autophagosome formation rather than reduces the lysosomal degradation activity.

### 3.7 Vimentin Knockdown Inhibits PI3K/AKT Signaling Pathway Activation to Increase Autophagy

**FIGURE 7:**
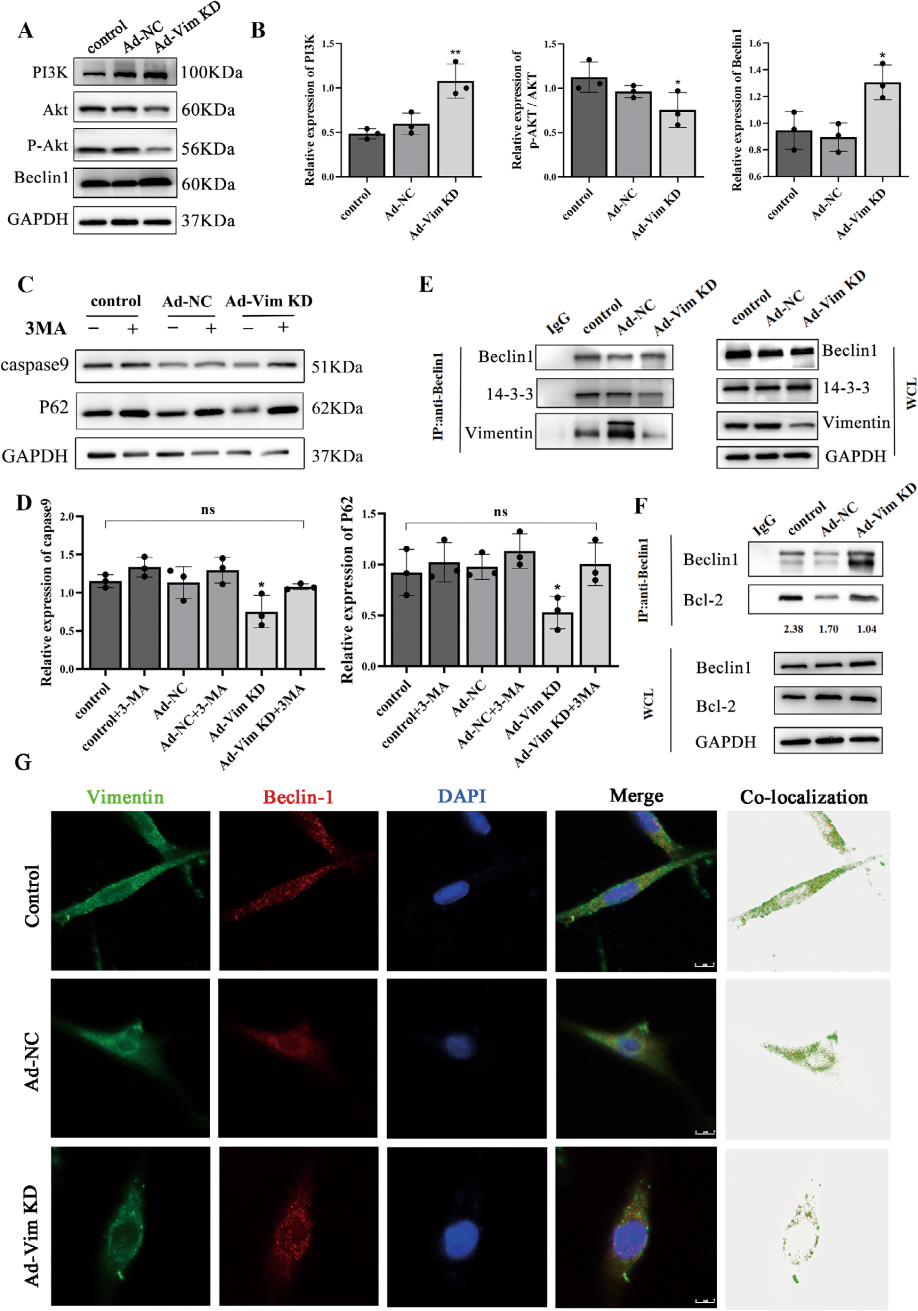
Vimentin knockdown inhibits PI3K/AKT signaling pathway activation to increase autophagy. (A) Representative Western blot results of PI3K, AKT, P‐AKT, Beclin1, GAPDH were used as an internal control and (B) relative quantitative analysis. (C) Representative Western blot results of apoptosis‐associated proteins caspase9, autophagy substrate proteins P62 in the presence or absence of 3‐MA, and (D) relative quantitative analysis. (E)CO‐Immunoprecipitation of vimentin, 14–3‐3 with endogenous Beclin1 in PC12 cells. (F) CO‐Immunoprecipitation of Bcl‐2 with endogenous Beclin1 in PC12 cells. (G) Representative images of vimentin and Beclin1 co‐localization.*p < 0.05, **p < 0.01, versus control.

Akt‐mediated phosphorylation can inhibit or enhance the activity of target proteins, thus regulating many types of downstream pathways. Western blot analysis revealed a significantly lower ratio of p‐Akt to Akt in the Ad‐Vim KD group compared to the control group. Additionally, immunoprotein blotting experiments demonstrated a notable increase in PI3K expression following vimentin knockdown (Figure 7A,B). To determine whether the anti‐apoptotic effects on SCI brought about by vimentin knockdown arise through changes in autophagy levels caused by affecting PI3K formation. Based on adenoviral infection, cells were treated with the autophagy inhibitor 3‐MA (Figure 7C,D). Vimentin knockdown significantly activated autophagic flux by down‐regulating the expression of autophagy substrate P62, whereas compared with Ad‐Vim KD, Ad‐Vim KD + 3‐MA treated resulted in the marked accumulation of p62. In addition, vimentin knockdown decreased the caspase9 expression level, which was up‐regulated by 3MA treatment.

Meanwhile, immunoprecipitation results demonstrated that the binding of endogenous Beclin1 to 14‐3‐3 and vimentin was significantly reduced in the Ad‐Vim KD group, when compared with the control group (Figure 7E), and the binding of endogenous Beclin1 to Bcl‐2 was decreased after vimentin knockdown (Figure 7F). Meanwhile, the co‐localization of Beclin1 with vimentin in cells was also significantly attenuated, and Beclin1 expression was significantly increased (Figure 7G). These results demonstrated vimentin knockdown could allow more Beclin1 dissociate from the cytoskeleton. Notably, Beclin1 was able to form Class III PI3K and activate PI (3)P to promote the formation of membrane‐independent structures in the early stages of autophagy. Suggesting that Beclin1 dissociated from the cytoskeleton as well as from the BH3 structure is likely to bind to Vps34 to form a Class III PI3K, which promotes autophagosome formation and enhances the level of cellular autophagy. In addition, Bcl‐2 dissociated from Beclin1 can exert its anti‐apoptotic effect and inhibit apoptosis.

## 4 Discussion

SCI is a severe disease of the central nervous system with a complex regeneration process. Following SCI, the local injury site develops a microenvironment characterized by ischemia, hypoxia, and inflammatory infiltration. In this context, the apoptosis of a large number of neurons is the primary cause of neuronal death and functional impairment.

Vimentin is a cytoskeletal protein belonging to the intermediate filament family. Under normal physiological conditions, vimentin is involved in maintaining cell integrity, while under various pathological conditions, it performs complex biological functions, thus exerting multiple physiological effects. Since vimentin is typically expressed primarily during development and is re‐expressed during astrocyte proliferation, most studies have focused on the relationship between vimentin and glial scar formation following SCI [15, 16]. Inhibition of vimentin expression effectively reduces neuroglial scar formation and promotes spinal cord axon regeneration [13]. Additionally, vimentin can clear local damaged organelles and misfolded proteins by regulating the autophagic level in neural stem cells, thereby restoring protein homeostasis. In this study, we found that vimentin knockdown significantly downregulated the expression of apoptotic proteins and reduced the apoptosis rate of neurons. These data indicate that vimentin can exert a protective effect against apoptosis in neural cells.

Autophagy is an innate immune defense mechanism against internal and external stressors, and numerous studies have reported a close relationship between autophagy and apoptosis [18, 19, 20]. The relationship between autophagy and apoptosis can be regulated by inhibiting their cross‐talk, and there is a tight interaction between the two in neurotrauma diseases, sharing multiple regulatory molecules and jointly influencing the fate of organelles and the entire cell [21, 22, 23]. However, the exact role of autophagy in SCI is controversial. On one hand, SCI leads to defects in autophagic flux, and the activation of autophagy can alleviate SCI and contribute to the recovery of neurological function [24]. On the other hand, overactivation of autophagy may lead to cell death, particularly in the later stages of SCI, which is detrimental to neural cells. Therefore, promoting autophagy in the early stages of SCI is beneficial for spinal cord repair, for the following reasons. Firstly, in the early stages of SCI, promoting autophagy can help clear damaged cellular components and reduce oxidative stress and inflammatory responses, thereby reducing cell death [25]. Secondly, early promotion of autophagy can facilitate the reconstruction, regeneration, and repair of cells by eliminating, degrading, and digesting damaged, denatured, aged, or dysfunctional cells, providing raw materials for cellular recycling [26]. In summary, the benefits of promoting autophagy in the early stages of SCI mainly lie in reducing cell death, inflammatory responses, and promoting cellular repair and functional recovery, which are vital for the recovery from SCI. In this study, we found that vimentin RNAi adenovirus transplantation significantly promoted the increase of autophagy‐related proteins LC3BII and Beclin‐1 protein levels and the decrease of p62 protein levels in spinal cord tissues. Moreover, at the cellular level, it was verified that the increase in autophagy caused by vimentin knockdown was mainly due to the increase in the formation of autophagosomes rather than the decrease in autophagic degradation activity, implying that the inhibition of vimentin expression could promote autophagic flow. Additionally, 3MA's inhibition of autophagy partially reversed the neuronal apoptosis induced by vimentin knockdown. Our results indicate that the activation of autophagy following vimentin treatment reverses neuronal apoptosis to some extent, but the specific mechanism requires further investigation.

Previous studies have shown that Beclin1, as an essential protein for the formation of autophagosomes and a response intermediate in the autophagy pathway, interacts with other autophagy‐associated proteins to form multiple complex signaling pathways that directly or indirectly affect autophagy and tumor feedback regulation. Beclin1 is able to act as a target of Akt phosphorylation, generating a binding site for 14‐3‐3 proteins into the binding vimentin regulates autophagy [27]. While Akt, as a PI3K/AKT signaling pathway protein, is closely related to autophagy and plays a key role in many mechanisms such as apoptosis, angiogenesis, as well as cell proliferation and migration to homeostasis in vivo, it is often over‐activated after SCI, which further exacerbates the SCI [17, 28, 29]. In this study, vimentin knockdown after SCI was able to effectively inhibit Akt phosphorylation, allowing more Beclin1 to be converted to the free state, reducing Beclin1 binding to 14‐3‐3 and vimentin, and favoring the interactions with Vps34, a lipase related to autophagy nucleation, which promotes autophagy. Meanwhile, COIP results showed that the binding between Beclin1 and Bcl‐2 was also reduced, and free Bcl‐2 exerted its own anti‐apoptotic effect to prevent apoptosis. Finally, after the addition of 3MA, a selective inhibitor of PI3K, the elevated autophagy level brought about by vimentin knockdown was successfully reversed, and the anti‐apoptotic effect was also successfully reversed.

To conclude, our research results indicate that after SCI, the knockdown of vimentin inhibits the interaction among vimentin, Beclin1, and 14‐3‐3, leading to more free Beclin1 and increased formation of PI3K. At the same time, the binding between Beclin1 and Bcl‐2 is also reduced, which enhances neuronal autophagy and inhibits apoptosis. Ultimately, the effects of vimentin knockdown improve the prognosis after SCI, suggesting that vimentin has therapeutic potential for SCI and could serve as an effective target for treatment. However, there are some limitations to our study. Firstly, we have not yet determined the specific upstream regulatory mechanisms of vimentin. Additionally, it remains to be further clarified whether vimentin can affect the autophagy of endogenous neural stem cells after SCI, thereby influencing the resting state exit process and participating in the repair of SCI.

## Author Contributions

Hongfu Wu and Xiaojun Cui designed research; Jie Zhao, Kangzhen Chen, Tao Wang, Tao He, Liji Chen and Xianxiu Qiu performed research; Xiaomin Zhang, Tao He, Jiahong Chen analyzed data; Kangzhen Chen, Jie Zhao and Hongfu Wu wrote the paper.

## Ethics Statement

The experiment was approved by the Institutional Animal Care and Use Committee of Guangdong Medical University, China.

## Conflicts of Interest

The authors declare no conflicts of interest.

## Supporting information


**Figure S1.** Vimentin knockdown inhibits neuronal proliferation and migration in vitro (A) EdU assay for the effect of vimentin inhibition on PC12 cell proliferation, scale bar = 200 μm. (B) EdU proliferation rate statistics. (C) Light microscopic observation of PC12 cell scratch healing assay, scale bar = 500 μm. (D) Statistics of PC12 cell fusion rate after 12 h and 24 h of scratch. **p < 0.01, ***p < 0.001, ****p < 0.0001, versuscontrol.

Data S1.

## Data Availability

The data that support the findings of this study are available on request from the corresponding author. The data are not publicly available due to privacy or ethical restrictions.
