# Supplementary figures and images for: Vimentin Inhibits Neuronal Apoptosis After Spinal Cord Injury by Enhancing Autophagy
Source: CNS Neurosci Ther. 2025 Jan 3;31(1):e70200. doi: 10.1111/cns.70200 (PMC11702385; doi:10.1111/cns.70200)

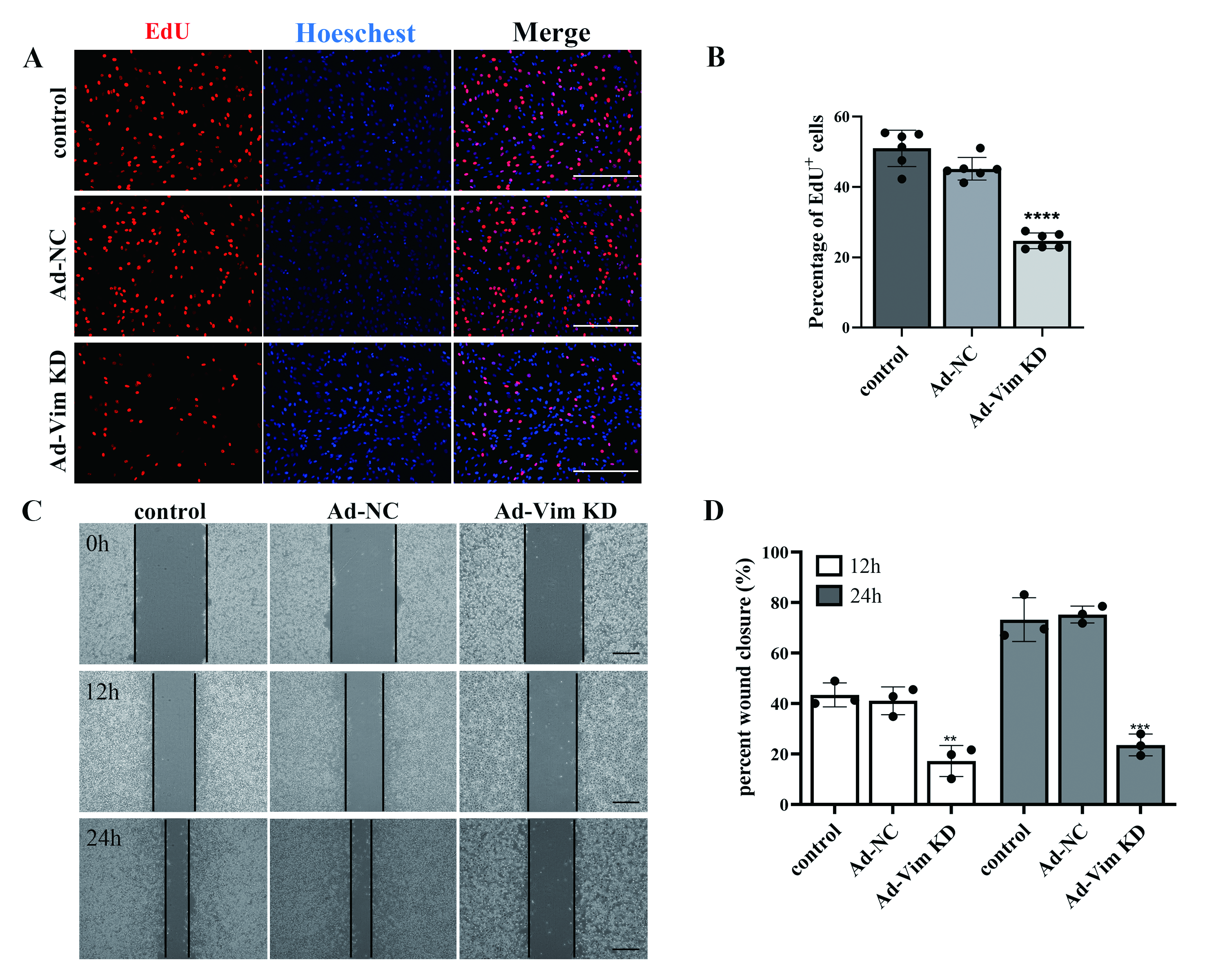

Supplement: Supplementary file 1 — Figure S1. Vimentin knockdown inhibits neuronal proliferation and migration in vitro (A) EdU assay for the effect of vimentin inhibition on PC12 cell proliferation, scale bar = 200 μm. (B) EdU proliferation rate statistics. (C) Light microscopic observation of PC12 cell scratch healing assay, scale bar = 500 μm. (D) Statistics of PC12 cell fusion rate after 12 h and 24 h of scratch. **p < 0.01, ***p < 0.001, ****p < 0.0001, versuscontrol. [file CNS-31-e70200-s002.tif]

# Full unedited blot

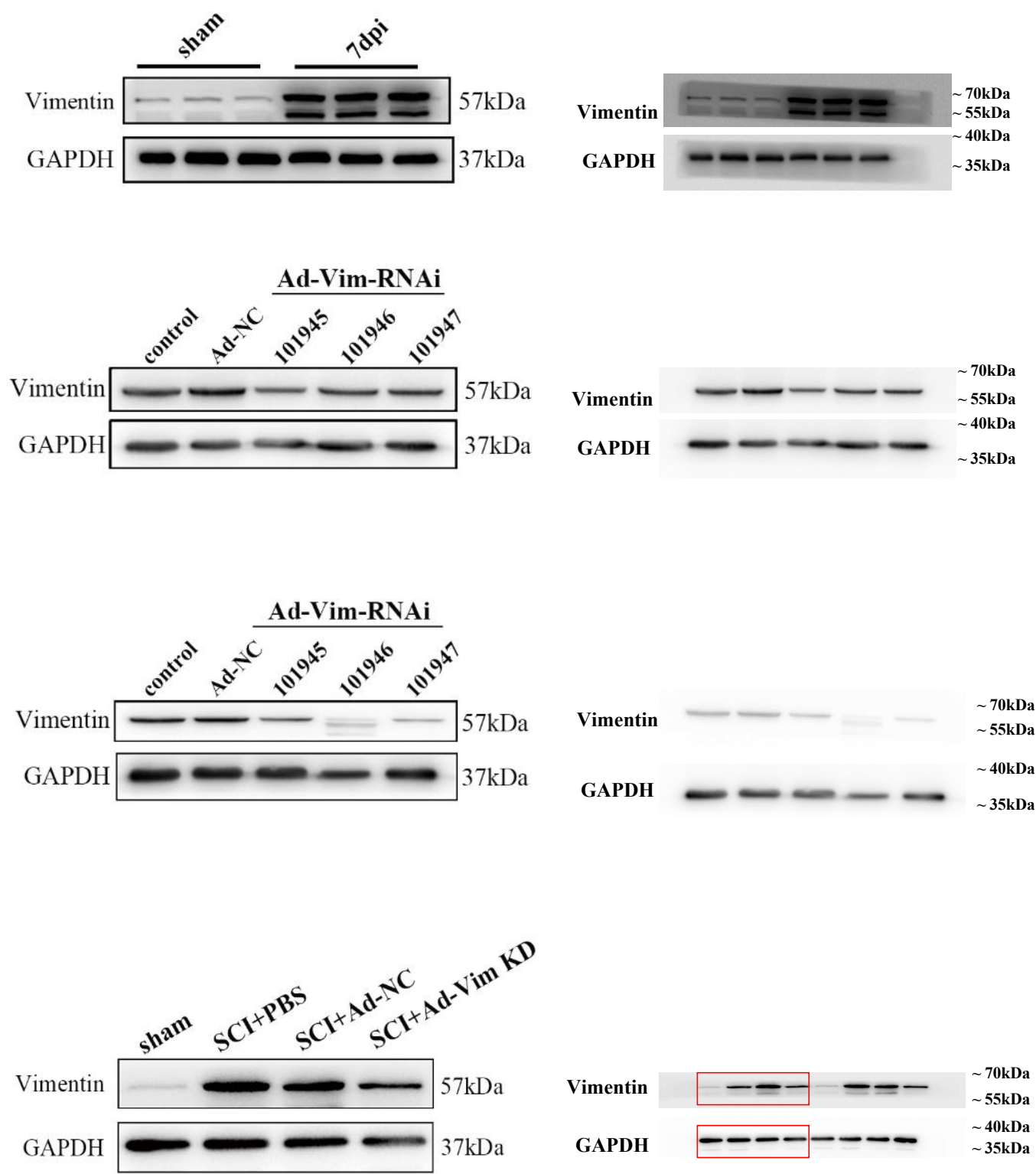

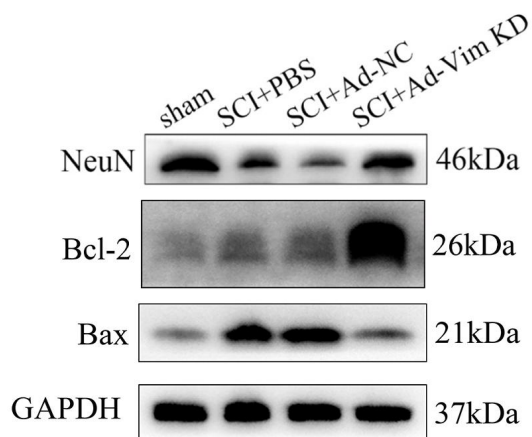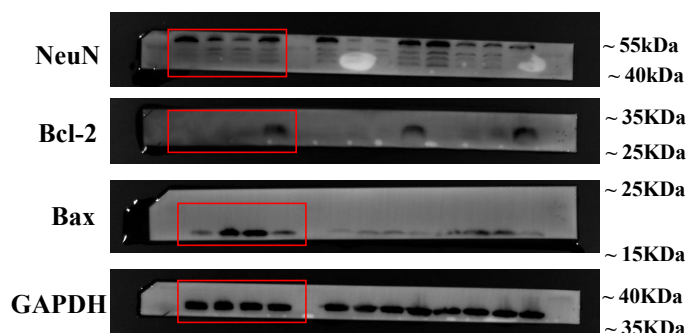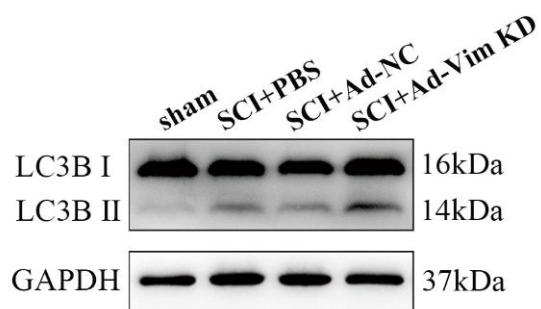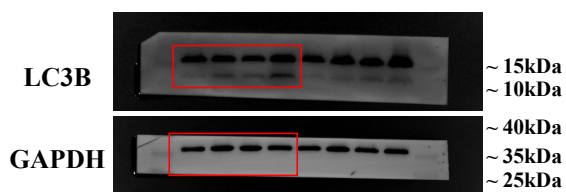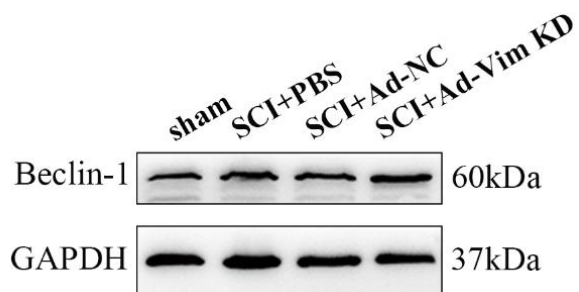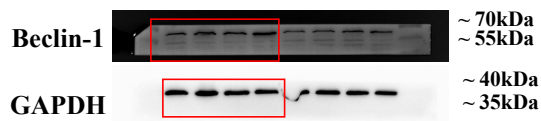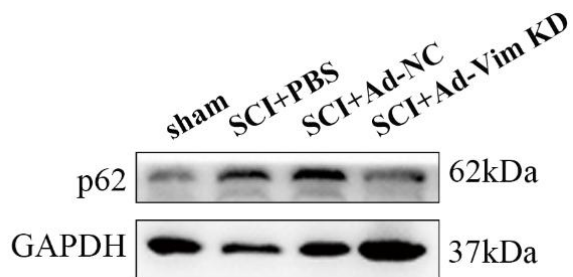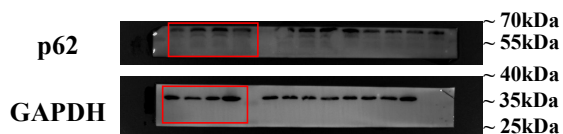

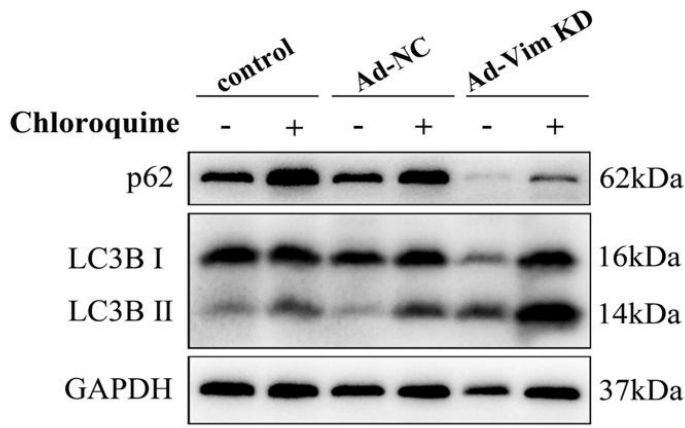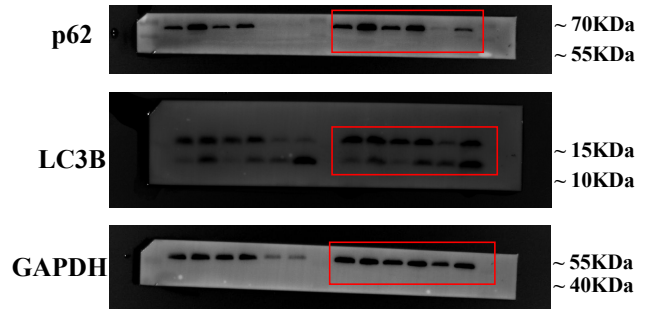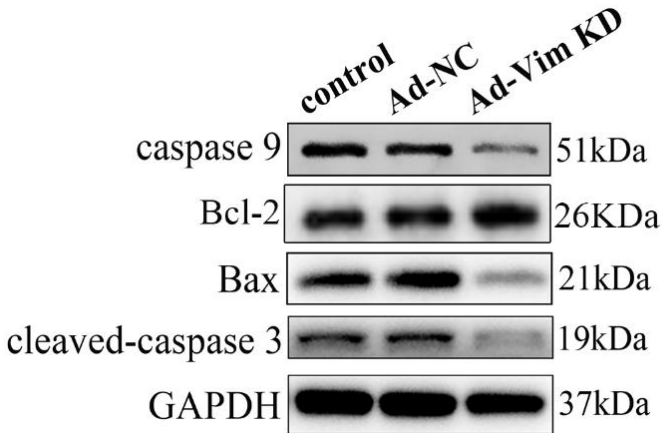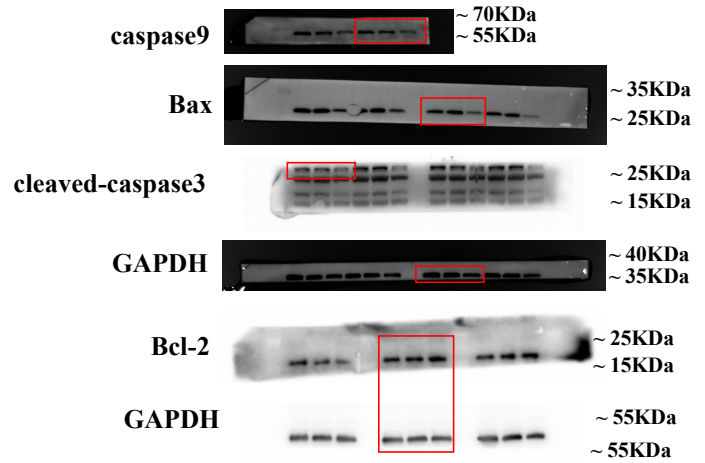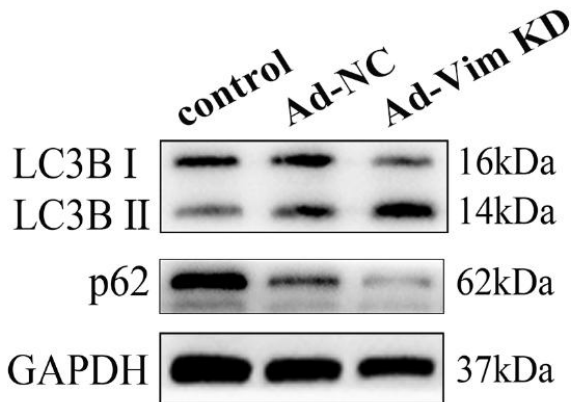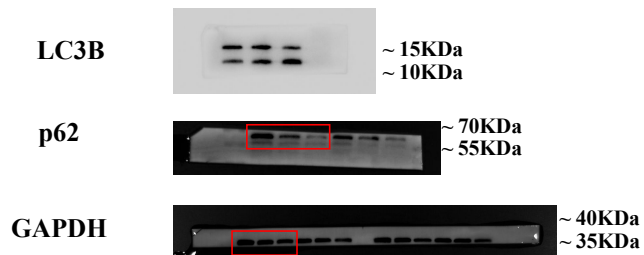

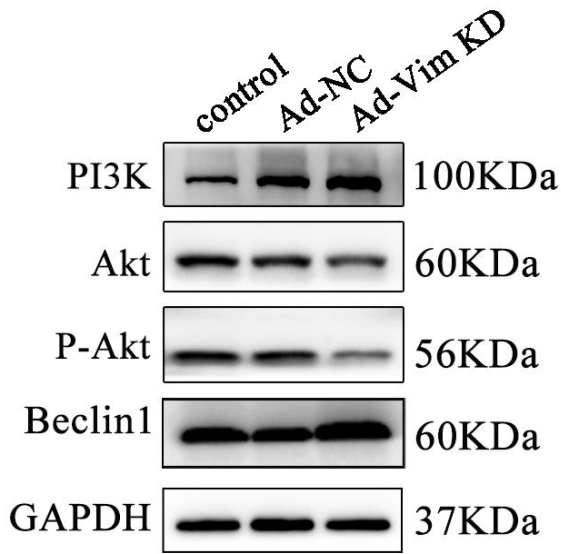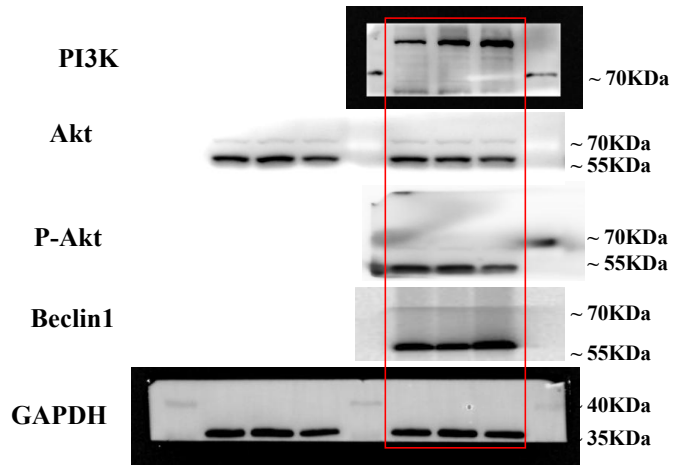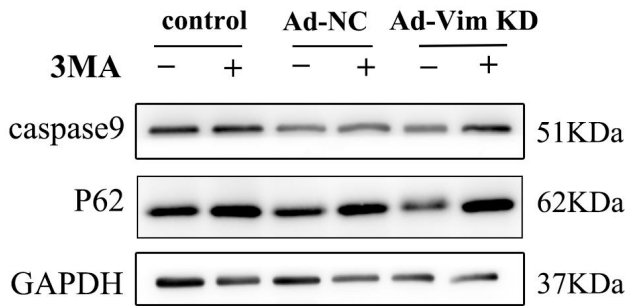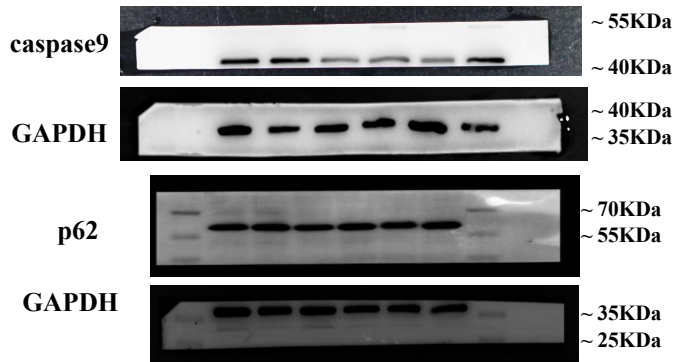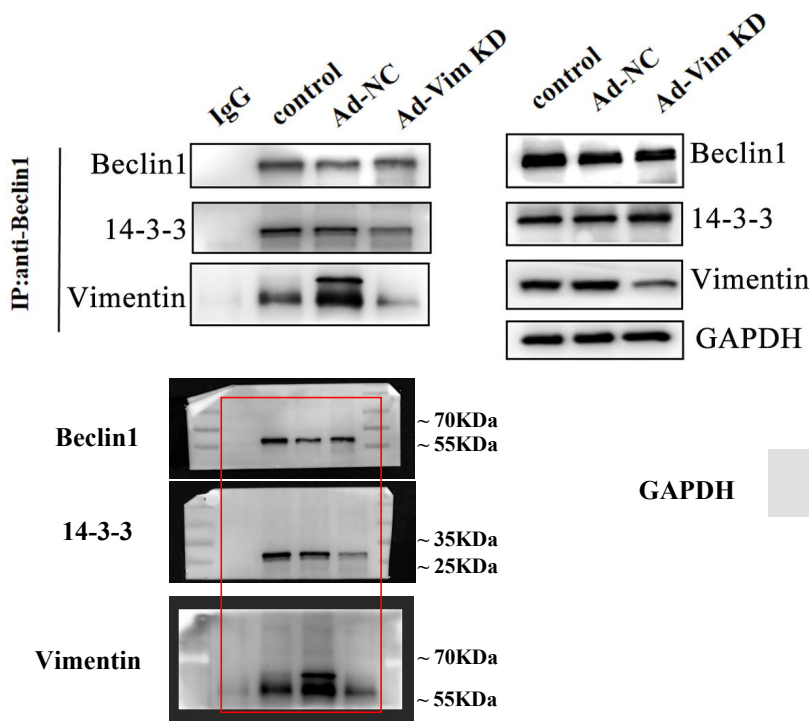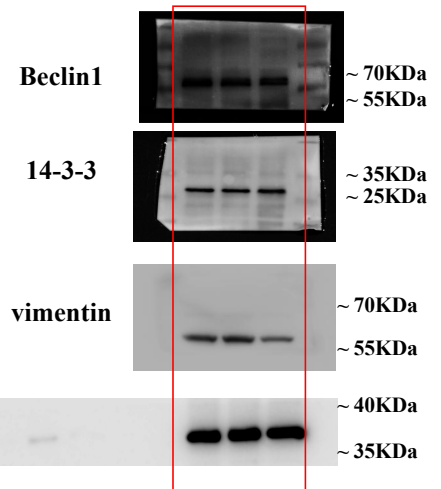

**F**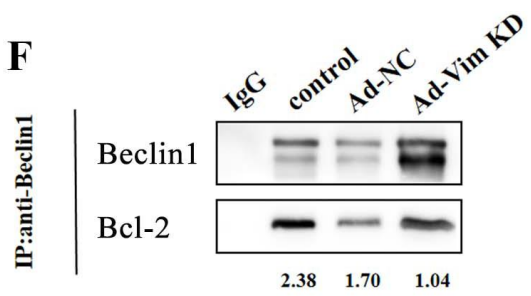**WCL**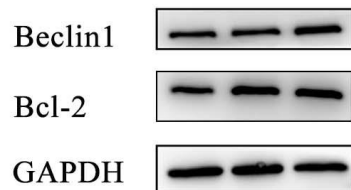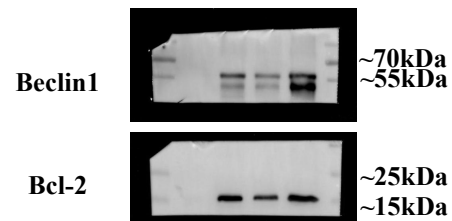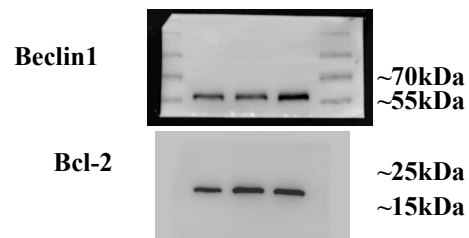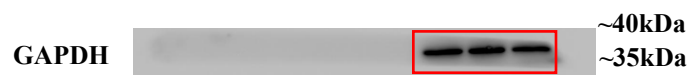

Supplement: Supplementary file 2 — Data S1. [file CNS-31-e70200-s001.pdf]
